# Optical Coherence Tomography-Guided Versus Angiography-Guided PCI in Moderate-to-Severe Calcified Coronary Lesions: A Systematic Review and Meta-Analysis of Randomized Trials

**DOI:** 10.3390/diagnostics16091317

**Published:** 2026-04-28

**Authors:** Hesham E. Mawar, Maryam Baamer, Azzam A. Althagafi, Ahmad G. Alghamdi, Moudi Aleidi, Reem S. Alzahrani, Abdulrahman Alnamlah, Maya F. Bokhari, Amjaad Batawi, Mohammed F. Gholam, Saad Al Bugami

**Affiliations:** 1 College of Medicine, King Saud bin Abdulaziz University for Health Sciences, Jeddah 22384, Saudi Arabia; baamer20188@ksau-hs.edu.sa (M.B.); 421120175@ksau-hs.edu.sa (A.A.A.); 421120012@ksau-hs.edu.sa (A.G.A.); aleidi20195@ksau-hs.edu.sa (M.A.); 421220096@ksau-hs.edu.sa (R.S.A.); 421120102@ksau-hs.edu.sa (A.A.); 421220188@ksau-hs.edu.sa (M.F.B.); batawi20018@ksau-hs.edu.sa (A.B.); gholamm@ksau-hs.edu.sa (M.F.G.); 2 King Abdullah International Medical Research Center, Jeddah 22384, Saudi Arabia; bogamisa@mngha.med.sa; 3 Department of Cardiology, King Faisal Cardiac Center, Ministry of National Guard Health Affairs, Jeddah 21423, Saudi Arabia

**Keywords:** coronary artery calcification, coronary artery disease, optical coherence tomography, percutaneous coronary intervention

## Abstract

**Background**: Moderate-to-severe coronary calcification is associated with worse outcomes following percutaneous coronary intervention (PCI). We aimed to assess the safety and efficacy of optical coherence tomography (OCT) compared with conventional angiography in PCI guidance of moderate-to-severe calcified coronary artery lesions. **Methods**: Multiple databases were systematically searched for outcomes of OCT- versus angiography-guided PCI in calcified lesions. Study selection and data extraction were conducted in accordance with the PRISMA guidelines. The primary endpoint was target vessel failure (TVF), a composite of cardiac death, target vessel myocardial infarction (TV-MI), and ischemia-driven target vessel revascularization (ID-TVR). Secondary endpoints included clinical (i.e., TVF components, stent thrombosis, and 30-day major adverse cardiovascular events [MACEs]), imaging, and procedural outcomes. **Results**: Four randomized controlled trials involving 3186 participants were included. Compared with angiography, OCT was associated with a significant reduction in TVF (risk ratio [RR] = 0.66; 95% confidence interval [CI]: 0.52–0.82), cardiac death (RR = 0.39; 95% CI: 0.22–0.70), TV-MI (RR = 0.63; 95% CI: 0.42–0.94), and stent thrombosis (RR = 0.24; 95% CI: 0.08–0.72). However, there were no significant changes in ID-TVR (RR = 0.77; 95% CI: 0.55–1.08) or 30-day MACEs (RR = 0.50; 95% CI: 0.16–1.61). Most procedural outcomes varied across studies and showed significant heterogeneity. **Conclusions**: OCT-guided PCI was associated with better clinical outcomes compared with angiography-guided PCI in this patient population. However, larger randomized trials are needed to confirm these results.

## 1. Introduction

Coronary artery disease is one of the leading causes of morbidity and mortality worldwide [1,2]. Coronary lesions are classified as either calcified or non-calcified lesions during percutaneous coronary intervention (PCI) [3]. Non-calcified lesions contain lipid-rich necrotic cores and fibrous caps, and they are associated with lower rates of major adverse cardiac events (MACEs) [4,5]. In contrast, calcified lesions have calcium deposition in the arterial wall. This characteristic results in increased arterial stiffness, reduced compliance, and greater resistance to balloon dilation [6]. These factors increase PCI procedural complexity, which can lead to malapposition, suboptimal stent expansion, increased procedural complications, and both short- and long-term MACEs [7,8,9]. Also, PCI of heavily calcified lesions is known to be clinically challenging. Moderate-to-severe calcified lesions constitute approximately one third to half of all PCI cases according to modern registries [10,11]. They are associated with poor clinical outcomes, including higher rates of stent failure and an increased need for repeat revascularization, when compared to non-calcified lesions [7,8,9]. Therefore, precise lesion characterization and optimized PCI strategies are essential for improving outcomes in patients with moderate-to-severe calcified coronary disease [6].

Optical coherence tomography (OCT) is an imaging modality in which near-infrared light produces high-resolution cross-sectional images of tissue microstructure. Its axial resolution of 10–15 μm allows detailed characterization of the superficial vessel wall, offering greater clarity than intravascular ultrasound (IVUS). Intravascular OCT provides enhanced evaluation of coronary artery structure and has the potential to improve outcomes during PCI. Additionally, it allows the measurement of calcium arc, thickness, and length since OCT light can penetrate calcium. These measurements help guide lesion preparation before stent implantation in calcified lesions. OCT also provides essential information for optimizing stent placement and accurate assessment of stent strut apposition to the arterial wall, helping reduce stent-related complications [12,13]. OCT transformed the diagnostic imaging across multiple specialties by overcoming depth and contrast limitations of conventional modalities and enabling early disease detection, precise structural characterization, and monitoring of therapeutic response [14].

Coronary angiography remains the standard guide for PCI although it provides two-dimensional views that limit the accuracy of the evaluation of plaques and vessel characteristics [15,16]. Angiography is effective in detecting coronary artery calcification when the calcium is highly eccentric (>180°), longer than 6 mm, and superficial. Conversely, when eccentricity is lower, calcification is shorter, or calcium is deep, diagnostic accuracy drops below 50%, causing underestimation of calcium [17]. These limitations have led to the adoption of intravascular imaging such as IVUS and OCT, which offer enhanced anatomical assessment and better PCI guidance [16].

Recent randomized clinical trials and meta-analyses have demonstrated that OCT-guided PCI reduces MACEs, target vessel failure (TVF), and stent thrombosis in complex coronary lesions [18,19]; however, evidence specifically on the moderate-to-severe calcified subgroup remains limited. Current guidelines, including the European Society of Cardiology (2024) and the American Heart Association (2025) [20,21], support the use of intravascular imaging with a Class I indication for PCI guidance in complex coronary lesions, particularly left main disease, bifurcations, and long lesions. However, these recommendations are largely derived from trials enrolling heterogeneous populations of complex lesions [20,21].

Although one network meta-analysis compared intravascular imaging with angiography across complex coronary lesions, all subgroups, including moderate-to-severe calcified lesions, were assessed only for MACEs, and the analysis did not include the recently published ECLIPSE trial [22,23]. Subgroup analyses from the ILUMIEN IV trial and the CALIPSO trial suggest that OCT guidance improves TVF, minimal stent area (MSA), and stent apposition in this subgroup [24,25]. These studies, however, are limited by sample size. In addition, no systematic review and meta-analysis has yet evaluated clinical, imaging, and procedural outcomes exclusively in patients with moderate-to-severe calcified coronary lesions. Therefore, this study aimed to compare OCT-guided PCI to angiography-guided PCI in the treatment of moderate-to-severe calcified coronary lesions, evaluating the clinical, imaging, and procedural outcomes.

## 2. Materials and Methods

### 2.1. Registration

Our study was conducted in accordance with the Preferred Reporting Items for Systematic Reviews and Meta-Analyses (PRISMA) 2020 statement and Cochrane Handbook of Systematic Reviews and Meta-Analyses [26,27]. The completed PRISMA checklist is provided in Table S1. The study protocol was prospectively registered in the International Prospective Register of Systematic Reviews (PROSPERO) database under the number CRD420251184947. This study was reviewed and approved by the Institutional Review Board of the King Abdullah International Medical Research Center (Protocol No. NRJ26/035/1) and was granted exemption due to its design as a systematic review and meta-analysis of published data. In addition, informed consent was not required.

### 2.2. Search Strategies

Several keywords including “Optical Coherence Tomography”, “Percutaneous Coronary Intervention”, “Coronary Artery Calcification”, and “complex coronary lesion” were used to comprehensively search the following databases: (1) MEDLINE via PubMed, (2) Web of Science, (3) Scopus, (4) EMBASE via Ovid, (5) Google Scholar, (6) Cochrane Central Register of Controlled Trials (CENTRAL), (7) online trial register ClinicalTrials.gov. The databases were searched from inception to 12 November 2025 with no restrictions on the study period or sample size. A detailed description of the search strategy can be found in Table S2.

### 2.3. Screening and Selection of Studies

Two reviewers (M.A. and R.S.A.) independently screened the titles and abstracts through Rayyan. Full-text articles were then independently assessed eligibility by A.A.A. and R.A., and a third reviewer (H.E.M.) resolved any disagreements. Only randomized controlled trials enrolling adult participants (≥18 years) who underwent PCI for moderate-to-severe calcified coronary lesions were included. In studies enrolling mixed lesion populations, data were extracted specifically from subgroups with moderate-to-severe calcified lesions when reported separately. Eligible studies were required to provide a direct comparison of any clinical, imaging, or procedural outcomes between OCT-guided and angiography-guided stent implantation. No language restrictions were applied, and conference abstracts were considered eligible. For Google Scholar, only the first 200 studies sorted by relevance were screened. Moreover, the evaluated clinical outcomes consisted of TVF, MACEs, and stent thrombosis. The examined imaging outcomes included MSA, malapposition, and edge dissection. Procedural outcomes included procedure and fluoroscopy duration, radiation dose, contrast volume, total stent length, number of post-dilation balloons, and the maximum pressure of inflation. When multiple publications or substudies reported overlapping populations, we selected the report with the most complete or most recent dataset. We excluded studies that used other intravascular imaging modalities (e.g., IVUS-guided PCI) where a direct comparison between OCT and angiography was not distinguished. In addition, studies that involved non-calcified or mildly calcified lesions where data for moderate-to-severe calcification cannot be separated were also excluded.

The primary endpoint of this meta-analysis was prospectively changed from MACEs to TVF prior to any data extraction or analysis, as documented in the registered PROSPERO protocol. This amendment was made because most eligible trials reported TVF as their primary clinical composite, which makes TVF the most consistently available and uniformly reported outcome across studies. We defined TVF as a composite of cardiac death, target vessel myocardial infarction (TV-MI), and ischemia-driven target vessel revascularization (ID-TVR), and it was assessed at the longest follow-up period. The remainder of the previously discussed outcomes were considered as secondary endpoints in our meta-analysis. All the endpoints were extracted from each trial and assessed on an intention-to-treat basis.

### 2.4. Data Collection and Extraction

Two independent reviewers (A.G.A. and M.A.) extracted each study’s data via a standardized data extraction sheet. The extracted data included the study characteristics (e.g., author, study name, and year of publication), baseline patient characteristics (e.g., age, sex, and medical history), and the aforementioned clinical, imaging, and procedural outcomes along with their corresponding definitions. Each independent reviewer searched the text, tables, and the supplementary appendix of each included study. For the CALIPSO trial [24], the 30-day MACE rate reported in the text was clarified by contacting the study authors. The confirmed number of events and total number of patients of this outcome were used in the pooled meta-analysis.

### 2.5. Risk-of-Bias Assessment

For the risk-of-bias assessment, two independent reviewers (A.A.A. and M.A.) assessed each eligible study using the Cochrane risk of bias tool for randomized trials (RoB 2) [28]. The evaluation included the following five domains: randomization process, deviations from intended interventions, missing outcome data, measurement of the outcomes, and selection of the reported result. A graphical summary of the results was created using robvis [29]. Reporting bias was planned to be assessed via visual inspection of funnel plots when at least 10 studies are included.

### 2.6. Certainty of Evidence Assessment

The certainty of evidence for each outcome was assessed using the Grading of Recommendations Assessment, Development, and Evaluation (GRADE) approach by two independent reviewers (A.A.A. and H.E.M.). The certainty of evidence was categorized as high, moderate, low, or very low depending on an overall evaluation across five domains: risk of bias, inconsistency, indirectness, imprecision, and publication bias. The tables of summaries of findings were generated via GRADEpro GDT [30,31].

### 2.7. Statistical Analysis

Data synthesis was performed using the Review Manager (RevMan) software version 5.4. Due to the anticipated clinical and procedural heterogeneity across studies, including differences in lesion preparation techniques (e.g., atherectomy or intravascular lithotripsy) and variation in clinical presentation and lesion complexity (e.g., acute coronary syndrome, left main disease, and chronic total occlusions), a random-effects model was used to calculate the pooled results of each individual study as risk ratios (RRs) for categorical outcomes (e.g., TVF, MACEs, and stent thrombosis) with 95% confidence intervals. The results of the continuous variables (e.g., MSA, stent expansion, and contrast volume) were pooled as mean difference (MD) or standardized mean difference (SMD), when appropriate, with 95% confidence intervals (CIs). To aid clinical interpretation of outcomes, absolute risk differences (ARDs) and the corresponding number needed to treat (NNT) were calculated from pooled event rates for selected outcomes with low event rates. Statistical heterogeneity was assessed by using I^2^, with values surpassing 50% deemed as significant heterogeneity. When appropriate, leave-one-out sensitivity analyses were performed to explore potential sources of heterogeneity and assess the robustness of the findings. The leave-one-out forest plot for TVF was generated using R (RStudio Version 2025.05.1+513) with the metafor package. No Hartung–Knapp or other variance adjustments were applied, and the default random-effects model in metafor was used.

For studies reporting continuous variables as median and interquartile range (IQR), we estimated the means and standard deviations using established methods using the following formulas [32,33]:

mean = (Q1 + median + Q3)/3(1)

standard deviation = IQR ÷ 1.35(2)

## 3. Results

### 3.1. Study Selection

**Figure 1:**
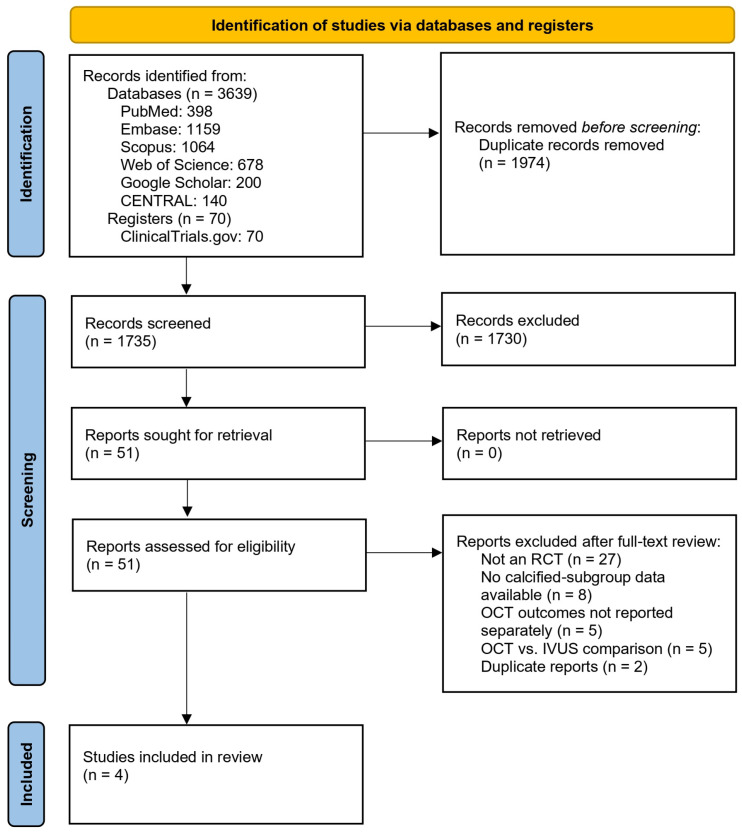
Study selection flowchart according to PRISMA statement [26].

A total of 3639 studies were identified from various databases, and 70 studies were found on clinical trial registries. Of those studies, 1974 duplicates were removed, and 1735 studies were screened. After screening by title and abstract, 1730 studies were excluded; thus, 51 studies were assessed for eligibility via full-text review. A total of 27 of these studies were excluded due to not being a randomized trial, 8 did not have calcified subgroup data, 5 did not report OCT outcomes separately, 5 compared OCT to IVUS without a direct comparison with angiography, and 2 were duplicate reports. Ultimately, 4 studies were included in this study [23,24,25,34] (Figure 1).

### 3.2. Characteristics of Included Studies

The baseline characteristics of the four included trials are summarized in Table 1, with additional details on angiographic and pre-PCI preparation techniques provided in Table S3. The included trials were published between 2023 and 2025. They had a total of 3186 patients divided between OCT and angiography-guided PCI (1626 in the OCT group and 1560 in the angio-PCI group). The studies included were all multicenter randomized controlled trials conducted across three continents (Europe, North America, and Asia). In two studies (CALIPSO and ECLIPSE) [23,24], all patients included underwent PCI for moderate-to-severe calcified coronary lesions, while the remaining two (ILUMIEN IV and OCTOBER) had a subgroup with moderate-to-severe calcifications [25,34]. To facilitate assessment of external validity, key trial characteristics that may influence generalizability, including moderate-to-severe calcification definitions, operator imaging experience, OCT protocol, and follow-up duration, are summarized in Table 2. The ECLIPSE trial let the operator use either IVUS or OCT according to their judgment [23], while the other three trials (CALIPSO, ILUMIEN IV, OCTOBER) adhered to using OCT-guided PCI for the intervention group of their patients [24,25,34]. The primary endpoints of the studies included TVF, MSA, and MACEs. In addition, outcome definitions were extracted from each trial’s published report where available and are summarized in Table S4.

### 3.3. Risk-of-Bias Assessment

Methodological quality assessment of the included trials via RoB 2 revealed that three of them had a low risk of bias. Only the CALIPSO trial revealed some concerns, specifically in the domain assessing missing outcome data [24]. All other domains had a low risk of bias across all studies (Figure S1). Reporting bias was not assessed as the current study included too few studies to perform funnel plot analysis.

### 3.4. Clinical Outcomes

**Figure 2:**
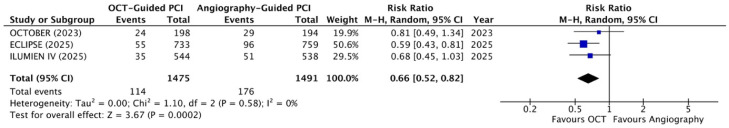
Forest plot of TVF comparing OCT- versus angiography-guided PCI in moderate-to-severe calcified lesions [23,25,34].

Three studies were included in the pooled analysis of TVF: the OCTOBER calcified-lesion subgroup, ILUMIEN IV, and ECLIPSE [23,25,34]. Across these studies, OCT-guided PCI was associated with a significantly lower risk of TVF compared with angiography guidance (RR = 0.66; 95% CI: 0.52–0.82; Figure 2), with no evidence of heterogeneity (I^2^ = 0%) and a weighted mean follow-up of 17.9 months. Because the OCTOBER trial used lesion-level components (target lesion myocardial infarction and target lesion revascularization) rather than vessel-level components (TV-MI and TVR) within its composite outcome [34], a sensitivity analysis excluding this trial was performed. The treatment effect remained consistent and statistically significant (RR = 0.62; 95% CI: 0.49–0.80; I^2^ = 0). This confirms that differences in endpoint definitions did not influence the overall findings (Figure S2).

**Figure 3:**
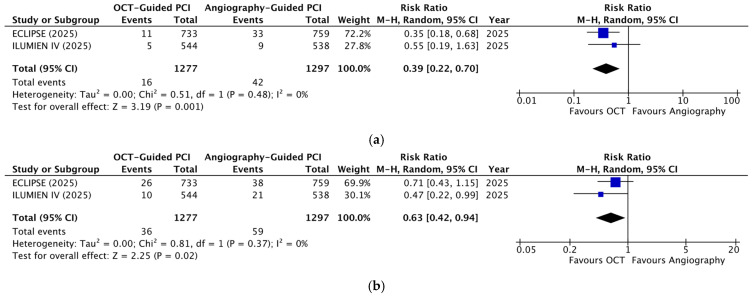

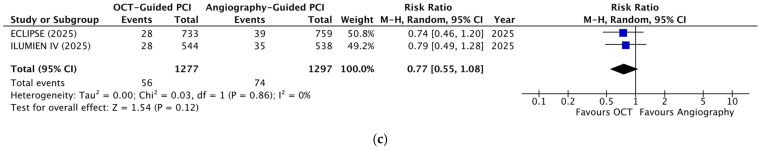
Forest plots of individual components of TVF comparing OCT- versus angiography-guided PCI in moderate-to-severe calcified lesions: (**a**) cardiac death; (**b**) TV-MI; (**c**) ID-TVR [23,25].

**Figure 4:**
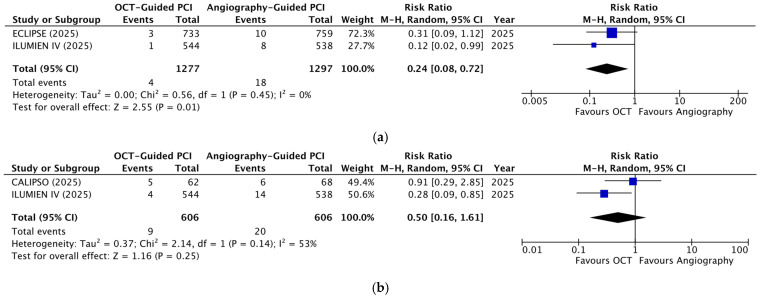
Forest plots of stent thrombosis and 30-day MACEs comparing OCT- versus angiography-guided PCI in moderate-to-severe calcified lesions: (**a**) stent thrombosis; (**b**) 30-day MACEs [23,24,25].

The analysis of cardiac death revealed a significant reduction in the OCT-guided group (RR = 0.39; 95% CI: 0.22–0.70; *p* = 0.001; Figure 3a) with no heterogeneity (I^2^ = 0%). TV-MI and stent thrombosis also demonstrated significant reductions with OCT guidance (RR = 0.63; 95% CI: 0.42–0.94; *p* = 0.02; Figure 3b and RR = 0.24; 95% CI: 0.08–0.72; *p* = 0.01; Figure 4a, respectively), with no heterogeneity observed for either outcome (I^2^ = 0%). ID-TVR did not show a statistically significant difference between the two groups (RR = 0.77; 95% CI: 0.55–1.08; *p* = 0.12; Figure 3c). The weighted mean follow-up period for cardiac death, TV-MI, ID-TVR, and stent thrombosis was 17 months. Analysis of 30-day MACEs also failed to demonstrate a statistically significant difference between the two groups (RR = 0.50; 95% CI: 0.16–1.61; *p* = 0.25; Figure 4b), with significant heterogeneity (I^2^ = 53%). Sensitivity analysis using a fixed-effects model did not alter these results.

To contextualize the clinical magnitude of the observed relative effects, the ARD and NNT were calculated for stent thrombosis and cardiac death given their low event rates. OCT-guided PCI was associated with an absolute risk reduction of 1.08% for stent thrombosis (ARD = −0.01; 95% CI: −0.02 to −0.001), corresponding to an NNT of 93. For cardiac death, the absolute risk reduction was 1.99% (ARD = −0.02; 95% CI: −0.04 to 0.00), corresponding to an NNT of 50. These findings suggest that approximately 50 and 93 patients would need to be treated with OCT guidance instead of angiography to prevent one cardiac death and one stent thrombosis event, respectively. However, these estimates should be interpreted cautiously because of their low number of events and the inclusion of only two trials in these analyses.

### 3.5. Imaging Outcomes

**Figure 5:**
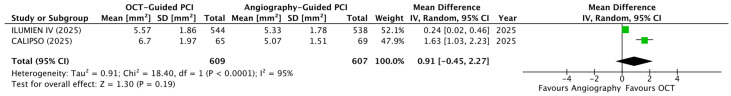
Forest plot of MSA comparing OCT- versus angiography-guided PCI in moderate-to-severe calcified lesions [24,25].

Two studies were included in the analysis of imaging outcomes, namely the CALIPSO and ILUMIEN IV studies [24,25]. MSA was greater in the OCT group; however, the effect was not statistically significant with a random-effect model (MD = 0.91; 95% CI −0.45–2.27; Figure 5) with high heterogeneity (I^2^ = 95%). Notably, sensitivity analysis using a fixed-effect model revealed a statistically significant effect favoring OCT (MD = 0.40; 95% CI: 0.20–0.61), and a leave-one-out analysis by excluding either study also yielded significant results. This, however, should be treated cautiously. Major malapposition in OCT guidance was greatly reduced when compared to angiography (RR = 0.57; 95% CI: 0.40–0.81; *p* = 0.002; I^2^ = 61%). Edge dissection was also reduced in the OCT group (RR 0.76; 95% CI: 0.65–0.89; *p* < 0.001) while exhibiting no heterogeneity (I^2^ = 0%) (Figure S3).

### 3.6. Procedural Outcomes

**Figure 6:**
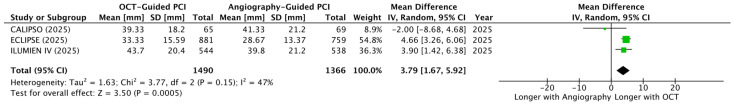
Forest plot of total stent length comparing OCT- versus angiography-guided PCI in moderate-to-severe calcified lesions [23,24,25].

Across the trials reporting procedural outcomes [23,24,25], OCT-guided PCI was associated with greater contrast use (MD = 25.68 mL; 95% CI: 11.46–39.89; *p* < 0.001; I^2^ = 77%) and longer procedure duration (MD = 11.93 min; 95% CI: 6.70–17.15; *p* < 0.001; I^2^ = 70%). Fluoroscopy time showed no significant pooled difference between the two modalities (MD = 1.69 min; 95% CI: 0.54–3.92; *p* = 0.14; I^2^ = 79%). Maximum inflation pressure similarly varied across trials, yielding no overall significant difference (MD = 0.00 atm; 95% CI: −1.32 to 1.33; *p* = 1.00; I^2^ = 95%). Stent length was significantly greater with OCT-guided PCI (MD = 3.79 mm; 95% CI: 1.67–5.92; *p* < 0.001; I^2^ = 47%; Figure 6), whereas the number of post-dilation balloons used (MD = 0.17; 95% CI: −0.12–0.46; *p* = 0.26; I^2^ = 74%) and radiation exposure (SMD = 0.10; 95% CI: −0.14–0.35; *p* = 0.42; I^2^ = 90%) did not differ significantly between groups. Excluding the CALIPSO trial in a leave-one-out sensitivity analysis reduced heterogeneity for contrast volume and fluoroscopy duration to 0%, and the effect remained significant (MD = 33.10 mL; 95% CI: 26.94–39.25; *p* < 0.001 and MD = 2.77 min; 95% CI: 1.87–3.67; *p* < 0.001, respectively) [24]. However, exclusion of the CALIPSO for the other procedural outcomes did not materially change the results. These outcomes, however, should be interpreted with caution as, except for stent length, heterogeneity was high across the pooled analyses (Figure S4).

### 3.7. Certainty of Evidence Assessment

A summary of findings for the assessed outcomes is presented in Table 3, and a detailed GRADE certainty of evidence provided in Tables S5–S7. In summary, most clinical outcomes had higher levels of certainty, followed by the imaging outcomes, and then the procedural outcomes. Among clinical outcomes, TVF and TV-MI had high certainty; cardiac death, ID-TVR, and stent thrombosis had moderate certainty; and 30-day MACEs had very low certainty. For imaging outcomes, edge dissection, major malapposition, and MSA were graded as having high, moderate, and low certainty, respectively. For procedural outcomes, total stent length was rated as high-certainty, while the remaining outcomes ranged from moderate- to very-low-certainty.

## 4. Discussion

In this meta-analysis, which included four randomized controlled trials, OCT-guided PCI showed better clinical outcomes in the treatment of patients with moderate-to-severe calcified coronary lesions. To elaborate, it significantly reduced TVF when compared to angiography-guided PCI during a weighted mean follow-up period of 17.9 months. A significant reduction in stent thrombosis was also observed with OCT-guided PCI with a weighted mean follow-up of 17 months. Among the three components of TVF, TV-MI and cardiac death were significantly reduced with OCT. However, these clinical benefits should be interpreted alongside the possible procedural tradeoffs. OCT-guided PCI was generally associated with longer procedure duration, higher contrast volume, and greater stent length across the included trials, reflecting the additional imaging steps required for lesion assessment and stent optimization. Therefore, the clinical value of OCT guidance lies in balancing improved lesion characterization and stent optimization against increased procedural complexity. To our knowledge, this is the first systematic review and meta-analysis evaluating the clinical, imaging, and procedural outcomes in moderate-to-severe calcified coronary lesions when comparing OCT to angiography.

Moderate-to-severe calcifications of the coronary vessels are associated with markedly worse clinical outcomes following PCI [7,8,9]. Two possible reasons that can explain this association are: (1) coronary calcification is an indicator of the severity of atherosclerosis, and (2) it is also associated with suboptimal post-PCI parameters, namely under expansion and increased incidence of edge dissections [8,9,35], and these parameters are associated with a greater incidence of MACEs and stent thrombosis [36,37]. Therefore, achieving optimal post-PCI results in heavily calcified lesions is particularly crucial. In this context, OCT can be valuable in the evaluation and management of heavily calcified lesions. First, OCT can accurately assess the severity and morphology of the calcifications, as opposed to conventional angiography, which usually underestimates the lesion [38,39]. Second, it can assess vessel preparation by detecting calcium fractures before implantation of the stent [39]. Third, it better assesses post-PCI parameters that might need optimization. Lastly, if needed, new bailout therapies may be used with OCT for under-expanded stents and correct them [35]. These mechanistic advantages are supported by the study’s imaging findings that revealed lower rates of edge dissection and major malapposition. Three of the trials included in this review supported these results indicating that stent implantation was improved with the guidance of OCT [23,24,25], which could explain the enhanced clinical outcomes observed in our population.

Prior to this study, a recent systematic review that evaluated the use of OCT versus angiography in the general coronary artery disease population showed that OCT was significantly associated with lower rates of adverse events and cardiovascular death; however, it revealed no significant difference in periprocedural MI or stent thrombosis [40]. In contrast to the present study, the OCCUPI trial demonstrated lower rates of TVR for the OCT group when compared to the angiography group in complex coronary lesions with lower cardiac deaths and MI rates as well [39]. This discrepancy could be explained by the differences in lesion characteristics in each study, as this study exclusively included moderate-to-severe calcified lesions, while the OCCUPI trial enrolled other complex lesions, including long lesions, bifurcations, small vessel disease, and visible thrombus with lower cardiac deaths and MI rates as well [41].

A recent network meta-analysis, which compared OCT, IVUS, and angiography in PCI guidance for complex coronary lesions, provides additional context for our findings [22]. Both OCT-guided PCI and IVUS-guided PCI were associated with significantly lower rates of MACEs, with no significant variation between OCT and IVUS, when compared to angiography-guided PCI in the subgroup analysis of calcified lesions [22]. Although this analysis supports the benefit of intravascular imaging over angiography, it is limited to the assessment of MACEs only and the inclusion of a smaller number of patients with calcified lesions compared to the present study. Our study helps in expanding these results by exclusively evaluating moderate-to-severe calcified lesions and by demonstrating a significant reduction in individual clinical endpoints, including cardiac death, TV-MI, and stent thrombosis. It also evaluated imaging and procedural outcomes associated with stent optimization.

The pooled MSA analysis demonstrated a directional benefit favoring OCT guidance; however, the effect did not reach statistical significance under the primary random-effects model and with significant heterogeneity (MD = 0.91; 95% CI: −0.45–2.27; I^2^ = 95%). The substantial heterogeneity observed in MSA in our study likely reflects differences in lesion complexity, calcium burden, and optimization criteria across trials. The high heterogeneity and low certainty of evidence found during the analysis limits the generalizability of our results. Despite the statistical uncertainty in the pooled MSA estimate, the consistent direction of effect in both trials and the significant reductions in major malapposition and edge dissection in the current study could provide convergent evidence that OCT guidance may improve the quality of stent implantation in calcified lesions. These imaging improvements are consistent with the prior literature demonstrating larger MSA and reduced malapposition with OCT guidance in both general coronary artery disease and calcified lesion populations [42,43], and could align mechanistically with the superior clinical outcomes observed in this meta-analysis.

With regard to procedural outcomes, in this research, there was a statistically significant increase in stent length in the OCT-guided PCI group. This is consistent with prior studies, such as ILUMIEN III, where greater stent length was associated with OCT usage. In prior studies, longer stent length is associated with significantly higher rates of MACEs and in-stent restenosis [44,45]. In the present review, however, the significant increase in total stent length did not translate into significant worsening in clinical outcomes during the 17.9 months weighted mean follow-up period. The increase in stent length with OCT guidance might be attributed to the ability of OCT to detect subtle proximal or distal lesions, thereby promoting more lesion coverage. Other procedural outcomes, such as procedure duration and contrast volume, were somewhat higher in the OCT group but exhibited significant heterogeneity amongst the groups. This is likely due to differences in OCT and angiography protocols, patients’ characteristics, and vessel preparation modalities across the trials. Nevertheless, this is also shown in the OCCUPI trial, where the OCT group uses more contrast and takes more time for the procedure [41]. The increased contrast volume observed with OCT-guided PCI is clinically relevant, particularly in patients with impaired renal function. Higher contrast exposure has been associated with an increased risk of contrast-induced acute kidney injury, which may adversely affect clinical outcomes following PCI [46]. Some recent studies have suggested that the increase in contrast volume in OCT-guided PCI may not increase the incidence of acute kidney injury compared with angiography-guided procedures [36]. However, evidence specifically in patients with heavily calcified lesions remains limited, and further studies are warranted to clarify the impact of OCT guidance on renal outcomes in this population.

Our study has some limitations. First, not all included trials exclusively enrolled patients with moderate-to-severe calcified coronary lesions. While CALIPSO and ECLIPSE studies specifically recruited such patients, OCTOBER and ILUMIEN IV provided calcified-lesion subgroup data from more extensive populations, namely complex bifurcation lesions and coronary lesions, respectively. This in turn may introduce potential indirectness. Moreover, as randomization was performed at the overall trial, randomization in these subgroup analyses is not guaranteed, which may increase risk of selection bias. However, excluding the OCTOBER or ILUMIEN IV trials in sensitivity analyses yielded consistent results, supporting the robustness of the findings. Second, several outcomes only included two trials, such as TV-MI, ID-TVR, cardiac death, 30-day MACEs, and stent thrombosis. This limited the ability to ensure robust sensitivity and subgroup analyses, although they were performed in some outcomes and resulted in limited statistical power for detecting rare clinical outcomes such as stent thrombosis and cardiac death. Third, the clinical outcome follow-up periods differed between the studies, ranging from one to two years. Fourth, the procedural outcomes in the analysis showed high heterogeneity across the trials, which could limit generalizability of findings in clinical practice. Fifth, due to the limited number of included studies, reporting bias could not be assessed. Therefore, the potential impact of unpublished or negative studies on the overall conclusions cannot be excluded. Finally, the variations in operator experience, OCT protocol, and definitions of TVF, MACEs, and malapposition among the studies could restrict the comparability of the pooled outcomes. For instance, the OCTOBER trial reported lesion-level components, whereas the other trials used vessel-level definitions. Although sensitivity analysis was done, pooling slightly heterogeneous composites may introduce conceptual bias.

## 5. Conclusions

OCT-guided PCI is associated with enhanced clinical and imaging-related parameters when compared with angiography-guided PCI in moderate-to-severe calcified coronary artery lesions, as evidenced by the lower incidences of TVF, TV-MI, cardiac death, stent thrombosis, and edge dissections. These findings are hypothesis-generating and support further dedicated randomized trials to confirm long-term benefit and define the optimal intravascular imaging strategy.

## Figures and Tables

**Table 1:** Characteristics of included studies.

Trial (Year)	Geography	Inclusion	Calcified Lesion Population	Total Patients, *n*	Moderate-to-Severe Calcified Lesions, *n*		Primary Endpoints	Definition of TVF/MACE
					OCT Group	Angiography Group		
OCTOBER (2023) [34]	Europe	True bifurcation lesions	Subgroup only	1201	198	194	MACE	MACE *: cardiac death, target-lesion myocardial infarction, or ischemia-driven target lesion revascularization
CALIPSO (2025) [24]	France	Moderate-to-severe calcified lesions	All patients	134	65	69	MSA	MACE: cardiovascular death, any myocardial infarction, or need for clinically driven reintervention on the target lesion
ECLIPSE (2025) [23]	US	Severe calcified lesions	All patients	2005	819	759	MSA, TVF	TVF: cardiac death, TV-MI, or ID-TVR
ILUMIEN IV (2025) [25]	US, Canada, Europe, Asia	Diabetic patients or complex lesions	Subgroup only	2487	544	538	MSA, TVF	MACE: cardiac death, TV-MI, or definite or probable stent thrombosis TVF: cardiac death, TV-MI, or ID-TVR

* In the OCTOBER trial, MACE definition is comparable to TVF definition used in ILUMIEN IV and ECLIPSE; therefore, MACE was treated as TVF in pooled analysis. Abbreviations: ID-TVR, ischemia-driven target vessel revascularization; MACE, major adverse cardiac event; MSA, minimal stent area; OCT, optical coherence tomography; TVF, target vessel failure; TV-MI, target vessel myocardial infarction.

**Table 2:** Key trial design features that may affect generalizability of included trials.

Trial (Year)	Definition of Moderate-to-Severe Calcified Lesions		Operator Experience	OCT Protocol (Pre-PCI)	OCT Protocol (Post-PCI)	Follow-Up Period
	Angiographic Severity	Angiographic Definition				
OCTOBER (2023) [34]	Moderate or severe	Radiopacity with contrast (moderate) or without contrast (severe) in the cardiac cycle before contrast injection	The majority of sites that enrolled patients were university centers, and most investigators had at least moderate experience with OCT guidance for PCI.	The protocol specified the timing of OCT scans, the checks and measurements to be obtained at each time point, the recommended optimizations, and the treatment goals.	The main treatment goals were optimal coverage of the coronary artery lesion with a stent, optimal stent expansion, and absence of stent malapposition or accidentally crushed or distorted stents.	2 years
CALIPSO (2025) [24]	Moderate or severe	Type B or C by Mintz classification	Not reported.	An initial OCT run was acquired, and the plaque preparation was guided by a predefined algorithm based on the maximal calcium arc extension for choice of the plaque.	Results were assessed by control OCT, and potential optimization steps were proposed according to predefined success criteria in terms of stent expansion, strut apposition, and presence of dissection based on the EAPCI consensus document.	30 days
ECLIPSE (2025) [23]	Severe	Radiopacities noted without cardiac motion involving both sides of the arterial wall, with total calcium length ≥ 15 mm extending into the target lesion	Not reported.	The protocol did not favor a specific IVI strategy or specify a target imaging goal; sites were asked to follow their usual, standard procedures for IVI use and stent optimization.	Selected sites were invited to participate in a voluntary OCT substudy to assess stent expansion in approximately 500 randomized patients.	1 year
ILUMIEN IV (2025) [25]	Moderate or severe	Radio-opacities seen only during the cardiac cycle (moderate) or without cardiac motion generally on both sides of the arterial lumen (severe)	Operators were experienced in OCT guided PCI and underwent mandatory training.	OCT was used to assess plaque morphology, stent size, and proximal and distal reference segments. Stent size was determined based on the mean external elastic lamina or the mean lumen-based vessel diameter.	OCT was performed, and post dilatation was conducted to ensure adequate stent expansion. Additional stenting was recommended in cases of major edge dissection or residual focal inflow/outflow disease in the reference segments.	30 days and 2 years

Abbreviations: EAPCI, European Association of Percutaneous Cardiovascular Interventions; IVI, intravascular imaging; OCT, optical coherence tomography; PCI, percutaneous coronary intervention.

**Table 3:** Summary of findings.

Outcomes	No. of Studies	Participants	Effect Estimate (95% CI)	*p*-Value	Heterogeneity (I^2^)
Clinical outcomes					
TVF	3	2966	RR 0.66 (0.52 to 0.82)	<0.001	0%
Cardiac death	2	2574	RR 0.39 (0.22 to 0.70)	0.001	0%
ID-TVR	2	2574	RR 0.77 (0.55 to 1.08)	0.12	0%
30-day MACEs	2	1212	RR 0.50 (0.16 to 1.61)	0.25	53%
Stent thrombosis	2	2574	RR 0.24 (0.08 to 0.72)	0.01	0%
TV-MI	2	2574	RR 0.63 (0.42 to 0.94)	0.02	0%
Imaging outcomes					
Edge Dissections	2	1214	RR 0.76 (0.65 to 0.89)	<0.001	0%
Major malapposition	2	1216	RR 0.57 (0.40 to 0.81)	0.002	61%
MSA (mm^2^)	2	1216	MD 0.91 (−0.45 to 2.27)	0.19	95%
Procedural outcomes					
Contrast volume (mL)	3	2708	MD 25.68 (11.46 to 39.89)	<0.001	77%
Fluoroscopy duration (min)	3	2708	MD 1.69 (−0.54 to 3.92)	0.14	79%
Maximum inflation pressure (atm)	3	2856	MD 0.00 (−1.32 to 1.33)	1.00	95%
No. of balloons	2	1216	MD 0.17 (−0.12 to 0.46)	0.26	74%
Procedure duration (min)	3	2708	MD 11.93 (6.70 to 17.15)	<0.001	70%
Radiation dose	2	2574	SMD 0.10 (−0.14 to 0.35)	0.42	90%
Total stent length (mm)	3	2856	MD 3.79 (1.67 to 5.92)	<0.001	47%

Abbreviations: ID-TVR, ischemia-driven target vessel revascularization; MACEs, major adverse cardiac events; MD, mean difference; MSA, minimal stent area; RR, risk ratio; SMD, standardized mean difference; TVF, target vessel failure; TV-MI, target vessel myocardial infarction; atm, standard atmosphere.

## Data Availability

The original contributions presented in this study are included in the article/Supplementary Materials. Further inquiries can be directed to the corresponding author.

## Supplementary Materials

The following supporting information can be downloaded at https://www.mdpi.com/article/10.3390/diagnostics16091317/s1, Table S1: PRISMA checklist; Table S2: Search strategy; Table S3: Baseline clinical, angiographic, and pre-PCI lesion preparation characteristics; Table S4: Stent thrombosis and imaging outcomes definitions; Table S5: GRADE assessment of the clinical outcomes; Table S6: GRADE assessment of the imaging outcomes; Table S7: GRADE assessment of the procedural outcomes; Figure S1: Risk-of-bias assessment of each eligible study by the Cochrane risk assessment tool 2; Figure S2: Leave-one-out sensitivity analysis forest plot of TVF; Figure S3: Forest plots of edge dissection and major malapposition comparing OCT- versus angiography-guided PCI in moderate-to-severe calcified lesions; Figure S4: Forest plots of procedural outcomes comparing OCT- versus angiography-guided PCI in moderate-to-severe calcified lesions.

## Abbreviations

The following abbreviations are used in this manuscript:

PCI	Percutaneous coronary intervention
MACE	Major adverse cardiovascular event
IVUS	Intravascular ultrasound
OCT	Optical coherence tomography
TVF	Target vessel failure
MSA	Minimal stent area
TV-MI	Target vessel myocardial infarction
ID-TVR	Ischemia-driven target vessel revascularization
