# Supplementary material for: Optical Coherence Tomography-Guided Versus Angiography-Guided PCI in Moderate-to-Severe Calcified Coronary Lesions: A Systematic Review and Meta-Analysis of Randomized Trials
Source: Diagnostics (Basel). 2026 Apr 28;16(9):1317. doi: 10.3390/diagnostics16091317 (PMC13163070; doi:10.3390/diagnostics16091317)
Supplement: Supplementary file 1 [file diagnostics-16-01317-s001.zip › diagnostics-4192934-supplementary.pdf]

Supplementary Material

Optical Coherence Tomography-Guided Versus Angiography-Guided PCI in Moderate-to-Severe Calcified Coronary Lesions: A Systematic Review and Meta-Analysis of Randomized Trials

Hesham E. Mawar, Maryam Baamer, Azzam A. Althagafi, Ahmad G. Alghamdi, Moudi Aleidi, Reem S. Alzahrani, Abdulrahman Alnamlah, Maya F. Bokhari, Amjaad Batawi, Mohammed F. Gholam, Saad Al Bugami

Table of Content

Table S1. PRISMA checklist 3
Table S2. Search strategy 6
Table S3. Baseline clinical, angiographic, and pre-PCI lesion preparation characteristics 7
Table S4. Stent thrombosis and imaging outcomes definitions 8
Table S5. GRADE assessment of the clinical outcomes 9
Table S6. GRADE assessment of the imaging outcomes 10
Table S7. GRADE assessment of the procedural outcomes 11
Figure S1. Risk-of-bias assessment of each eligible study by the Cochrane Risk Assessment Tool 2 12
Figure S2. Leave-one-out sensitivity analysis forest plot of TVF 13
Figure S3. Forest plots of edge dissection major malapposition 14
Figure S4. Forest plots of procedural outcomes 15

Table S1. PRISMA checklist

Section and Topic	Item #	Checklist item	Location where item is reported
**TITLE**			
Title	1	Identify the report as a systematic review.	Title
**ABSTRACT**			
Abstract	2	See the PRISMA 2020 for Abstracts checklist.	Abstract
**INTRODUCTION**			
Rationale	3	Describe the rationale for the review in the context of existing knowledge.	Introduction
Objectives	4	Provide an explicit statement of the objective(s) or question(s) the review addresses.	Introduction, last paragraph
**METHODS**			
Eligibility criteria	5	Specify the inclusion and exclusion criteria for the review and how studies were grouped for the syntheses.	Section 2.3. Screening and selection of studies
Information sources	6	Specify all databases, registers, websites, organisations, reference lists and other sources searched or consulted to identify studies. Specify the date when each source was last searched or consulted.	Section 2.2. Search strategies
Search strategy	7	Present the full search strategies for all databases, registers and websites, including any filters and limits used.	Table S2. Search strategy
Selection process	8	Specify the methods used to decide whether a study met the inclusion criteria of the review, including how many reviewers screened each record and each report retrieved, whether they worked independently, and if applicable, details of automation tools used in the process.	Section 2.3. Screening and selection of studies
Data collection process	9	Specify the methods used to collect data from reports, including how many reviewers collected data from each report, whether they worked independently, any processes for obtaining or confirming data from study investigators, and if applicable, details of automation tools used in the process.	Section 2.4. Data collection and extraction
Data items	10a	List and define all outcomes for which data were sought. Specify whether all results that were compatible with each outcome domain in each study were sought (e.g. for all measures, time points, analyses), and if not, the methods used to decide which results to collect.	Section 2.3. Screening and selection of studies

	10b	List and define all other variables for which data were sought (e.g. participant and intervention characteristics, funding sources). Describe any assumptions made about any missing or unclear information.	Section 2.3; Section 2.4.
Study risk of bias assessment	11	Specify the methods used to assess risk of bias in the included studies, including details of the tool(s) used, how many reviewers assessed each study and whether they worked independently, and if applicable, details of automation tools used in the process.	Section 2.5. Risk of bias assessment
Effect measures	12	Specify for each outcome the effect measure(s) (e.g. risk ratio, mean difference) used in the synthesis or presentation of results.	Section 2.7. Statistical analysis
Synthesis methods	13a	Describe the processes used to decide which studies were eligible for each synthesis (e.g. tabulating the study intervention characteristics and comparing against the planned groups for each synthesis (item #5)).	Section 2.4. Data collection and extraction
	13b	Describe any methods required to prepare the data for presentation or synthesis, such as handling of missing summary statistics, or data conversions.	Section 2,4; Section 2.7
	13c	Describe any methods used to tabulate or visually display results of individual studies and syntheses.	Sections 2.5–2.7
	13d	Describe any methods used to synthesize results and provide a rationale for the choice(s). If meta-analysis was performed, describe the model(s), method(s) to identify the presence and extent of statistical heterogeneity, and software package(s) used.	Section 2.7. Statistical analysis
	13e	Describe any methods used to explore possible causes of heterogeneity among study results (e.g. subgroup analysis, meta-regression).	Section 2.7. Statistical analysis
	13f	Describe any sensitivity analyses conducted to assess robustness of the synthesized results.	Section 2.7. Statistical analysis
Reporting bias assessment	14	Describe any methods used to assess risk of bias due to missing results in a synthesis (arising from reporting biases).	NA
Certainty assessment	15	Describe any methods used to assess certainty (or confidence) in the body of evidence for an outcome.	Section 2.6. Certainty of evidence assessment
**RESULTS**			
Study selection	16a	Describe the results of the search and selection process, from the number of records identified in the search to the number of studies included in the review, ideally using a flow diagram.	Section 3.1. Study selection; Figure 1

	16b	Cite studies that might appear to meet the inclusion criteria, but which were excluded, and explain why they were excluded.	Section 3.1. Study selection
Study characteristics	17	Cite each included study and present its characteristics.	Section 3.2. Characteristics of included studies
Risk of bias in studies	18	Present assessments of risk of bias for each included study.	Section 3.3. Risk of bias assessment
Results of individual studies	19	For all outcomes, present, for each study: (a) summary statistics for each group (where appropriate) and (b) an effect estimate and its precision (e.g. confidence/credible interval), ideally using structured tables or plots.	Figs. 2–6
Results of syntheses	20a	For each synthesis, briefly summarise the characteristics and risk of bias among contributing studies.	Section 3.3. Risk of bias assessment
	20b	Present results of all statistical syntheses conducted. If meta-analysis was done, present for each the summary estimate and its precision (e.g. confidence/credible interval) and measures of statistical heterogeneity. If comparing groups, describe the direction of the effect.	Sections 3.5–3.7; Figs. 2–6
	20c	Present results of all investigations of possible causes of heterogeneity among study results.	Sections 3.5–3.7
	20d	Present results of all sensitivity analyses conducted to assess the robustness of the synthesized results.	Sections 3.5–3.7
Reporting biases	21	Present assessments of risk of bias due to missing results (arising from reporting biases) for each synthesis assessed.	NA
Certainty of evidence	22	Present assessments of certainty (or confidence) in the body of evidence for each outcome assessed.	Section 3.4. Certainty of evidence assessment; tables S4–S6
**DISCUSSION**			
Discussion	23a	Provide a general interpretation of the results in the context of other evidence.	Discussion
	23b	Discuss any limitations of the evidence included in the review.	Discussion, paragraph 7
	23c	Discuss any limitations of the review processes used.	Discussion, paragraph 7
	23d	Discuss implications of the results for practice, policy, and future research.	Discussion, paragraph 8

OTHER INFORMATION			
Registration and protocol	24a	Provide registration information for the review, including register name and registration number, or state that the review was not registered.	Section 2.1. Registration
	24b	Indicate where the review protocol can be accessed, or state that a protocol was not prepared.	Section 2.1. Registration
	24c	Describe and explain any amendments to information provided at registration or in the protocol.	Section 2.3. Screening and selection of studies
Support	25	Describe sources of financial or non-financial support for the review, and the role of the funders or sponsors in the review.	End matter: Sources of funding
Competing interests	26	Declare any competing interests of review authors.	End matter: Declaration of competing interest
Availability of data, code and other materials	27	Report which of the following are publicly available and where they can be found: template data collection forms; data extracted from included studies; data used for all analyses; analytic code; any other materials used in the review.	End matter: Data availability statement

Table S2. Search strategy

Database	Search date	Search strategy	Results
**PubMed**	12/11/2025	(("optical coherence tomography"[tiab] OR OCT[tiab] OR "intravascular imaging"[tiab]) AND ("Percutaneous Coronary Intervention"[Mesh] OR "percutaneous coronary intervention"[tiab] OR PCI[tiab] OR "coronary stent*"[tiab] OR "coronary stenting"[tiab]) AND ("calcified lesion*"[tiab] OR "calcification"[tiab] OR "complex lesion*"[tiab] OR "complex coronary lesion*"[tiab]))	398
**Embase**	12/11/2025	TS = (("optical coherence tomography" OR OCT OR "intravascular imaging") AND ("percutaneous coronary intervention" OR PCI OR "coronary stent*" OR "coronary stenting") AND ("calcified lesion*" OR "calcification" OR "calcified" OR “complex lesion” OR “complex coronary lesion”))	1159
**Scopus**	12/11/2025	(TITLE-ABS-KEY("optical coherence tomography" OR OCT OR "intravascular imaging") AND TITLE-ABS-KEY("percutaneous coronary intervention" OR PCI OR "coronary stent*" OR "coronary stenting") AND TITLE-ABS-KEY("calcified lesion*" OR " calcification" OR "calcified" OR "calcif*" OR “complex lesion” OR “complex coronary lesion”))	1064
**Web of Science**	12/11/2025	TS = (("optical coherence tomography" OR OCT OR "intravascular imaging") AND ("percutaneous coronary intervention" OR PCI OR "coronary stent*" OR "coronary stenting") AND ("calcified lesion*" OR "calcification" OR "calcified" OR “complex lesion” OR “complex coronary lesion”))	678
**Google Scholar**	12/11/2025	("optical coherence tomography" OR OCT OR "intravascular imaging") AND ("percutaneous coronary intervention" OR PCI OR (coronary NEXT stent*) OR (coronary NEXT stenting)) AND ((calcified NEXT lesion*) OR calcified OR calcification OR (complex NEXT lesion) OR (complex NEXT coronary NEXT lesion))	200
**ClinicalTrials.gov**	12/11/2025	Condition or disease: calcified lesion OR calcification OR complex lesion OR complex coronary lesion<br>Other terms: "optical coherence tomography" OR OCT OR “intravascular imaging	70
**Cochrane**	15/3/2026	#1 MeSH descriptor: [Percutaneous Coronary Intervention] explode all trees<br>#2 MeSH descriptor: [Tomography, Optical Coherence] explode all trees<br>#3 (Intravascular imaging):ti,ab,kw<br>#4 Complex coronary lesion):ti,ab,kw OR (Complex lesion):ti,ab. kw (Word variations have been searched)<br>#5 (Calcified):ti,ab,kw<br>#6 ("percutaneous coronary intervention"):ti,ab,kw<br>#7 ("optical coherence tomography"):ti,ab,kw<br>#8 (#1 OR #6)<br>#9 (#2 OR #3 OR #7)<br>#10 (#4 OR #5)<br>#11 (#8 AND #9 AND #10)	140

Table S3. Baseline clinical, angiographic, and pre-PCI lesion preparation characteristics

Trial	Age (year)	Male	Hypertension	Diabetes	Dyslipidemia	ACS	LM	CTO	Atherectomy	Lithotripsy	Balloon	Cutting, scoring, or specialty balloon used
**OCTOBER* (2023)**	66.4/66.2	78.8/79.0	70.3/74.5	17.2/16.1	76.0/78.4	45.0/46.6 †	NR	Excluded	NR	NR	NR	NR
**CALIPSO (2025)**	72.0/74.0	80.0/83.0	71.0/62.0	37.0/39.0	86.0/88.0	Excluded	6.0/7.0‡	NR	19/26	46/12	NC balloon: 35/61	NR
**ECLIPSE (2025)**	70.0/71.0	74.8/70.0	87.7/91.3	40.5/45.5	89.2/86.1	16.5/20.4	0.3/1.3	NR	48.5/48.8	Discouraged	93.6/89.9	14.9/6.0
**ILUMIEN IV (2025)**	67.3/67.0	77.2/76.2	72.2/76.4	41.5/39.6	64.9/68.4	56.1/51.1	Excluded	5.7/5.0	Advanced lesion preparation performed¶: 17.5/9.9			

Data are present as mean or proportion of OCT-guidance group/angiography guidance group (%).
*Data reflect the main study population as insufficient data were reported for the calcified-lesion subset.
†Including staged procedure after acute myocardial infarction
‡Distal left main; ostial left main artery lesions were excluded
¶Advanced lesion preparation in ILUMIEN IV includes pre-stent treatment with a cutting or scoring balloon, atherectomy, lithotripsy, or laser.
Abbreviations: ACS, acute coronary syndrome; CTO, chronic total occlusion; LM, left main; NR, not reported; OCT, Optical Coherence Tomograph; PCI, percutaneous coronary intervention

Table S4. Stent thrombosis and imaging outcomes definitions

Trial (year)	Stent thrombosis	Edge dissections	Major malapposition	MSA
OCTOBER (2023) [34]	Stent thrombosis is categorized as acute, sub-acute, late and very late and as definite, probable and possible according to the ARC criteria.	NR	NR	NR
CALIPSO (2025) [24]	NR	NR	NR	Procedural success was defined as combination of outcomes including MSA greater than 4.5 mm^2
ECLIPSE (2025) [23]	NR	NR	NR	NR
ILUMIEN IV (2025) [25]	Definite or probable stent thrombosis was defined according to the modified ARC-2 criteria	Defined as ≥ 60° of the circumference of the vessel at site of dissection and ≥3 mm in length.	Malapposition is defined as stent struts clearly separated from the vessel wall without any tissue behind the struts with a distance from the adjacent intima of ≥ 0.2 mm and not associated with any side branch. Major malapposition is defined as malapposition associated with unacceptable stent expansion.	Adequate stent expansion defined as MSA ≥90% of the closest reference lumen area.

Abbreviations: ARC-2, Academic Research Consortium-2; MSA, minimal stent area; NR, not reported; OCT, optical coherence tomography.

Table S5. GRADE assessment of the clinical outcomes

Certainty assessment							No. of patients		Effect		Certainty	Importance
**No. of studies**	**Study design**	**Risk of bias**	**Inconsistency**	**Indirectness**	**Imprecision**	**Other considerations**	**OCT**	**angiography**	**Relative (95% CI)**	**Absolute (95% CI)**		
TVF												
**3**	randomised trials	not serious	not serious	not serious	not serious	none	114/1475 (7.7%)	176/1491 (11.8%)	**RR 0.62** (0.49 to 0.80)	**45 fewer per 1,000** (from 60 fewer to 24 fewer)	⊕⊕⊕⊕ High	CRITICAL
Cardiac death												
**2**	randomised trials	not serious	not serious	not serious	serious[b]	none	16/1277 (1.3%)	42/1297 (3.2%)	**RR 0.39** (0.22 to 0.70)	**20 fewer per 1,000** (from 25 fewer to 10 fewer)	⊕⊕⊕◯ Moderate[b]	CRITICAL
TV-MI												
**2**	randomised trials	not serious	not serious	not serious	not serious	none	36/1277 (2.8%)	59/1297 (4.5%)	**RR 0.63** (0.42 to 0.94)	**17 fewer per 1,000** (from 26 fewer to 3 fewer)	⊕⊕⊕⊕ High	CRITICAL
ID-TVR												
**2**	randomised trials	not serious	not serious	not serious	serious[a]	none	56/1277 (4.4%)	74/1297 (5.7%)	**RR 0.77** (0.55 to 1.08)	**13 fewer per 1,000** (from 26 fewer to 5 more)	⊕⊕⊕◯ Moderate[a]	CRITICAL
Stent thrombosis												
**2**	randomised trials	not serious	not serious	not serious	serious[b]	none	4/1277 (0.3%)	18/1297 (1.4%)	**RR 0.24** (0.08 to 0.72)	**11 fewer per 1,000** (from 13 fewer to 4 fewer)	⊕⊕⊕◯ Moderate[b]	CRITICAL
30-day MACE												
**2**	randomised trials	not serious	serious[c]	not serious	very serious[a,b]	none	9/606 (1.5%)	20/606 (3.3%)	**RR 0.50** (0.16 to 1.61)	**17 fewer per 1,000** (from 28 fewer to 20 more)	⊕◯◯◯ Very low[a,b,c]	CRITICAL

a. CI ranges from significant benefit with OCT to significant benefit with angiography
b. Low event count
c. Visual inspection of forest plot suggests some heterogeneity; $I^2 > 50\%$
Abbreviations: CI, confidence interval; RR, risk ratio; OCT, optical coherence tomography

Table S6. GRADE assessment of the imaging outcomes

Certainty assessment							No. of patients		Effect		Certainty	Importance
**No. of studies**	**Study design**	**Risk of bias**	**Inconsistency**	**Indirectness**	**Imprecision**	**Other considerations**	**OCT**	**Angiography**	**Relative (95% CI)**	**Absolute (95% CI)**		
MSA												
**2**	randomised trials	not serious	serious[a]	not serious	serious[b]	none	609	607	-	MD **0.91 mm² higher** (0.45 lower to 2.27 higher)	⊕⊕◯◯ Low[a,b]	IMPORTANT
Edge Dissection												
**2**	randomised trials	not serious	not serious	not serious	not serious	none	183/608 (30.1%)	240/606 (39.6%)	**RR 0.76** (0.65 to 0.89)	**95 fewer per 1,000** (from 139 fewer to 44 fewer)	⊕⊕⊕⊕ High	IMPORTANT
Major Malapposition												
**2**	randomised trials	not serious	serious[a]	not serious	not serious	none	131/609 (21.5%)	249/607 (41.0%)	**RR 0.57** (0.40 to 0.81)	**176 fewer per 1,000** (from 246 fewer to 78 fewer)	⊕⊕⊕◯ Moderate[a]	IMPORTANT

a. Visual inspection of forest plot suggests some heterogeneity; $I^2 > 50\%$
b. CI ranges from significant benefit with OCT to significant benefit with angiography
Abbreviations: CI**,** confidence interval; MD, mean difference; RR**,** risk ratio; OCT, optical coherence tomography

Table S7. GRADE assessment of the procedural outcomes

Certainty assessment							No. of patients		Effect	Certainty	Importance
**No. of studies**	**Study design**	**Risk of bias**	**Inconsistency**	**Indirectness**	**Imprecision**	**Other considerations**	**OCT**	**Angiography**	**Absolute (95% CI)**		
Procedure Duration											
**3**	randomised trials	not serious	serious[a]	not serious	not serious	none	1342	1366	MD **11.93 min higher** (6.7 higher to 17.15 higher)	⊕⊕⊕◯ Moderate[a]	IMPORTANT
Fluoroscopy Duration											
**3**	randomised trials	not serious	serious[a]	not serious	serious[b]	none	1342	1366	MD **1.69 min higher** (0.54 lower to 3.92 higher)	⊕⊕◯◯ Low[a,b]	IMPORTANT
Radiation Dose											
**2**	randomised trials	not serious	very serious[c]	not serious	serious[b]	none	1277	1297	SMD **0.1 higher** (0.14 lower to 0.35 higher)	⊕◯◯◯ Very low[b,c]	IMPORTANT
Contrast Volume											
**3**	randomised trials	not serious	serious	not serious	not serious	none	1342	1366	MD **25.68 mL higher** (11.46 higher to 39.89 higher)	⊕⊕⊕◯ Moderate	IMPORTANT
Total stent Length											
**3**	randomised trials	not serious	not serious	not serious	not serious	none	1490	1366	MD **3.79 mm higher** (1.67 higher to 5.92 higher)	⊕⊕⊕⊕ High	IMPORTANT
Number of Postdilation Balloons											
**2**	randomised trials	not serious	serious[a]	not serious	serious[b]	none	609	607	MD **0.17 higher** (0.12 lower to 0.46 higher)	⊕⊕◯◯ Low[a,b]	IMPORTANT
Maximum Inflation Pressure											
**3**	randomised trials	not serious	very serious[c]	not serious	serious[b]	none	1490	1366	MD **0 atm** (1.32 lower to 1.33 higher)	⊕◯◯◯ Very low[b,c]	IMPORTANT

a. $I^2 > 50\%$
b. Confidence interval ranges from significant benefit with OCT to significant benefit with angiography
c. $I^2 \geq 90\%$
Abbreviations: CI**,** confidence interval; MD, mean difference; SMD, standardized mean difference; OCT, optical coherence tomography

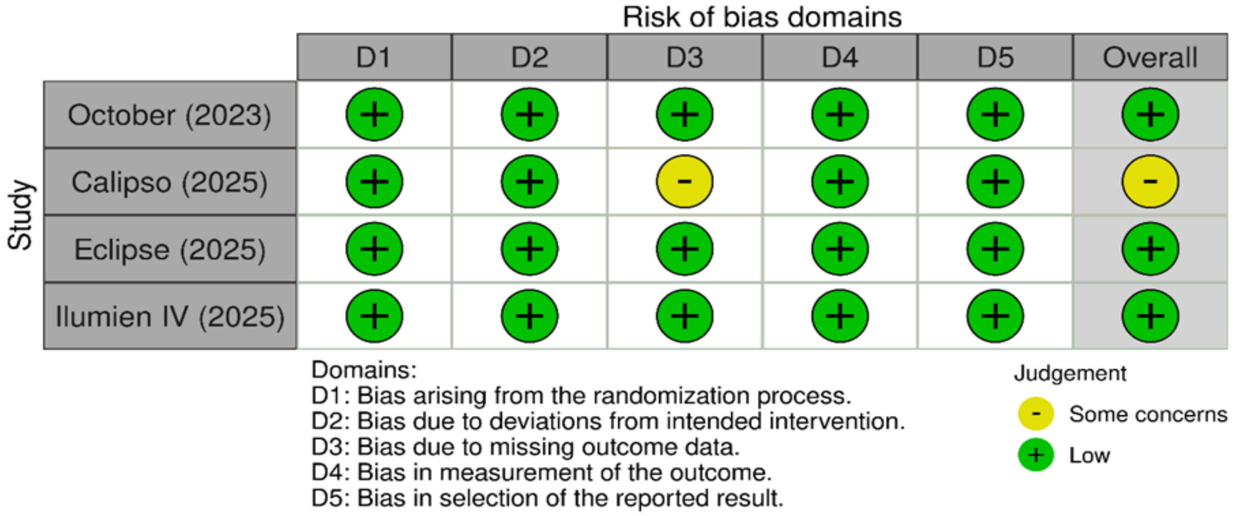

Figure S1. Risk-of-bias assessment of each eligible study by the Cochrane Risk Assessment Tool 2 [28]. The Robvis tool was used to generate this figure [29]

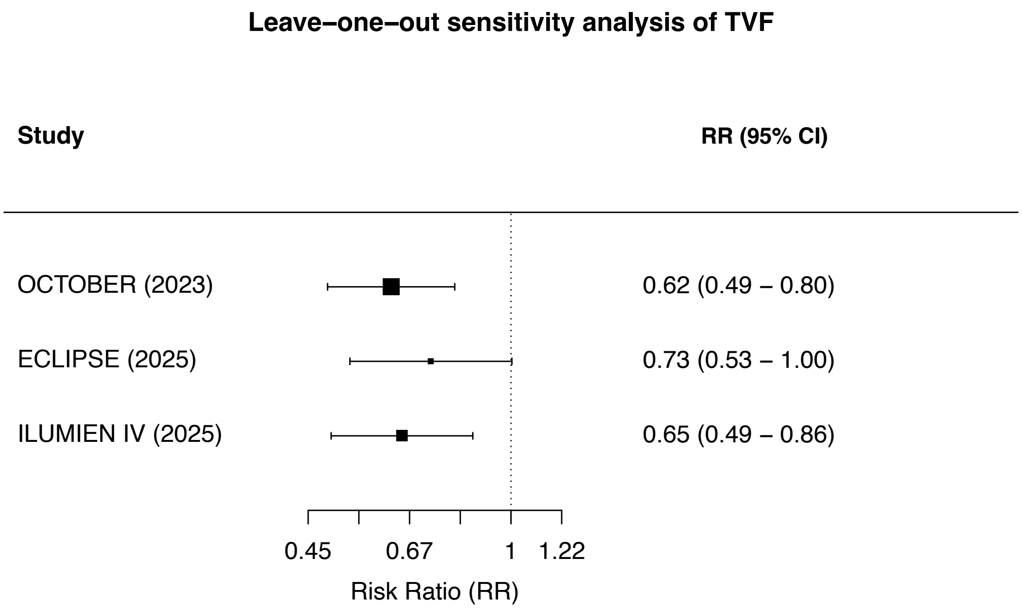

Figure S2. Leave-one-out sensitivity analysis forest plot of TVF. Leave-one-out sensitivity analysis was performed using R (R Foundation for Statistical Computing) with the metafor package

(**a**) Edge dissection

Study or Subgroup	OCT–Guided PCI Events	OCT–Guided PCI Total	Angiography–Guided PCI Events	Angiography–Guided PCI Total	Weight	Risk Ratio M–H, Random, 95% CI	Year
CALIPSO (2025)	16	65	28	69	9.3%	0.61 [0.36, 1.01]	2025
ILUMIEN IV (2025)	167	543	212	537	90.7%	0.78 [0.66, 0.92]	2025
**Total (95% CI)**		**608**		**606**	**100.0%**	**0.76 [0.65, 0.89]**	
Total events	183		240				

Heterogeneity: $Tau^2 = 0.00$; $Chi^2 = 0.83$, df = 1 (P = 0.36); $I^2 = 0\%$
Test for overall effect: Z = 3.43 (P = 0.0006)

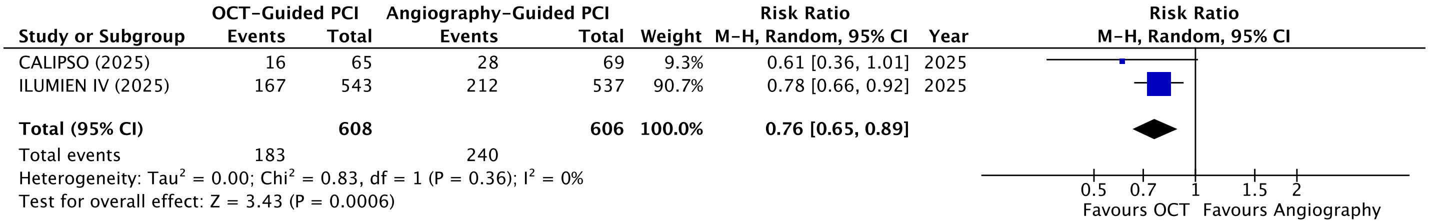

(**b**) Major malapposition

Study or Subgroup	OCT–Guided PCI Events	OCT–Guided PCI Total	Angiography–Guided PCI Events	Angiography–Guided PCI Total	Weight	Risk Ratio M–H, Random, 95% CI	Year
CALIPSO (2025)	23	65	34	69	38.0%	0.72 [0.48, 1.08]	2025
ILUMIEN IV (2025)	108	544	215	538	62.0%	0.50 [0.41, 0.61]	2025
**Total (95% CI)**		**609**		**607**	**100.0%**	**0.57 [0.40, 0.81]**	
Total events	131		249				

Heterogeneity: $Tau^2 = 0.04$; $Chi^2 = 2.57$, df = 1 (P = 0.11); $I^2 = 61\%$
Test for overall effect: Z = 3.11 (P = 0.002)

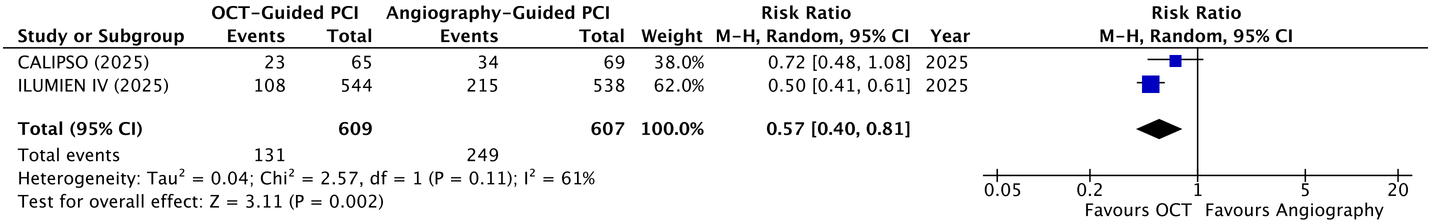

Figure S3. Forest plots of edge dissection and major malapposition comparing OCT- versus angiography-guided PCI in moderate-to-severe calcified lesions. (**a**) Edge dissection; (**b**) Major malapposition

(a) Contrast volume

Study or Subgroup	OCT-Guided PCI Mean [mL]	SD [mL]	Total	Angiography-Guided PCI Mean [mL]	SD [mL]	Total	Weight	Mean Difference IV, Random, 95% CI
CALIPSO (2025)	184.33	70.51	65	184.33	52.24	69	22.8%	0.00 [−21.11, 21.11]
ECLIPSE (2025)	178.33	74.28	733	145.67	76.5	759	40.4%	32.66 [25.01, 40.31]
ILUMIEN IV (2025)	230.4	88.6	544	196.5	85.2	538	36.8%	33.90 [23.54, 44.26]
**Total (95% CI)**			**1342**			**1366**	**100.0%**	**25.68 [11.46, 39.89]**

Heterogeneity: $Tau^2 = 114.97$; $Chi^2 = 8.74$, df = 2 (P = 0.01); $I^2 = 77\%$
Test for overall effect: Z = 3.54 (P = 0.0004)

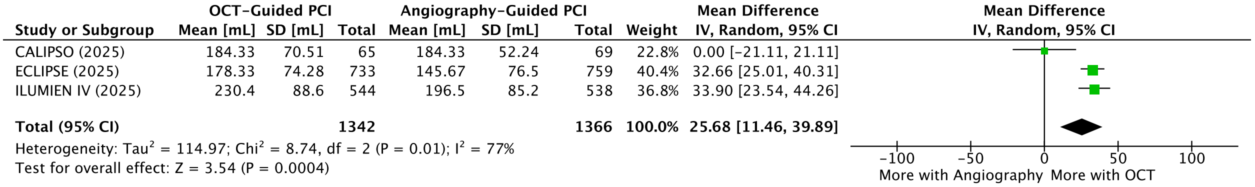

(b) Fluoroscopy duration

Study or Subgroup	OCT-Guided PCI Mean [min]	SD [min]	Total	Angiography-Guided PCI Mean [min]	SD [min]	Total	Weight	Mean Difference IV, Random, 95% CI	Year
CALIPSO (2025)	16.33	7.58	65	18.67	11.36	69	23.0%	−2.34 [−5.59, 0.91]	2025
ECLIPSE (2025)	20.23	11.29	733	17.7	10.03	759	40.6%	2.53 [1.45, 3.61]	2025
ILUMIEN IV (2025)	21.3	14.2	544	18	12.7	538	36.4%	3.30 [1.70, 4.90]	2025
**Total (95% CI)**			**1342**			**1366**	**100.0%**	**1.69 [−0.54, 3.92]**	

Heterogeneity: $Tau^2 = 2.88$; $Chi^2 = 9.42$, df = 2 (P = 0.009); $I^2 = 79\%$
Test for overall effect: Z = 1.49 (P = 0.14)

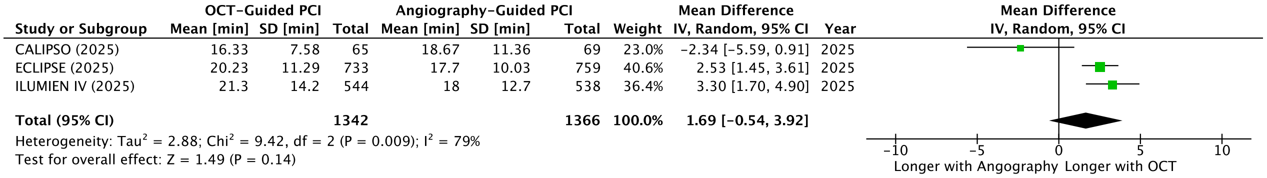

(c) Maximum inflation pressure

Study or Subgroup	OCT-Guided PCI Mean [atm]	SD [atm]	Total	Angiography-Guided PCI Mean [atm]	SD [atm]	Total	Weight	Mean Difference IV, Random, 95% CI	Year
CALIPSO (2025)	16	3.03	65	18	3.03	69	29.7%	−2.00 [−3.03, −0.97]	2025
ECLIPSE (2025)	19.1	3.8	881	18.8	3.5	759	35.2%	0.30 [−0.05, 0.65]	2025
ILUMIEN IV (2025)	20	3.1	544	18.6	3.3	538	35.1%	1.40 [1.02, 1.78]	2025
**Total (95% CI)**			**1490**			**1366**	**100.0%**	**0.00 [−1.32, 1.33]**	

Heterogeneity: $Tau^2 = 1.26$; $Chi^2 = 44.20$, df = 2 (P < 0.00001); $I^2 = 95\%$
Test for overall effect: Z = 0.01 (P = 1.00)

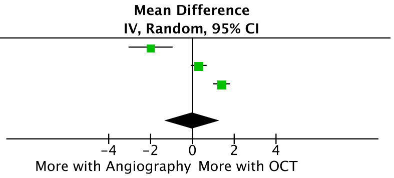

(d) number of used balloons

Study or Subgroup	Experimental Mean	SD	Total	Control Mean	SD	Total	Weight	Mean Difference IV, Random, 95% CI
CALIPSO (2025)	1.33	0.76	65	1.33	0.76	69	43.6%	0.00 [−0.26, 0.26]
ILUMIEN IV (2025)	1.8	1.2	544	1.5	1.3	538	56.4%	0.30 [0.15, 0.45]
**Total (95% CI)**			**609**			**607**	**100.0%**	**0.17 [−0.12, 0.46]**

Heterogeneity: $Tau^2 = 0.03$; $Chi^2 = 3.91$, df = 1 (P = 0.05); $I^2 = 74\%$
Test for overall effect: Z = 1.14 (P = 0.26)

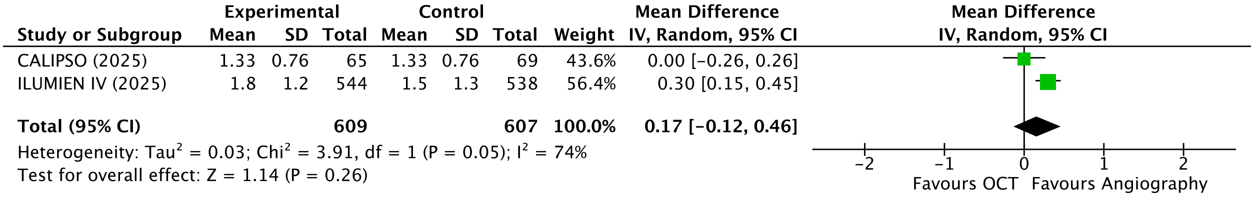

Figure S4. Forest plots of procedural outcomes comparing OCT- versus angiography-guided PCI in moderate-to-severe calcified lesions. (**a**) Contrast volume; (**b**) Fluoroscopy duration; (**c**) Maximum inflation pressure; (**d**) Number of used balloons; (**e**) Procedure duration; (**f**) Radiation dose

(**e**) Procedure duration

Study or Subgroup	OCT-Guided PCI Mean [min]	OCT-Guided PCI SD [min]	OCT-Guided PCI Total	Angiography-Guided PCI Mean [min]	Angiography-Guided PCI SD [min]	Angiography-Guided PCI Total	Weight	Mean Difference IV, Random, 95% CI	Year
CALIPSO (2025)	67	27.29	65	64	27.26	69	19.6%	3.00 [−6.24, 12.24]	2025
ECLIPSE (2025)	64.67	31.2	733	52.33	27.48	759	43.6%	12.34 [9.35, 15.33]	2025
ILUMIEN IV (2025)	67.7	38	544	51.5	37.8	538	36.8%	16.20 [11.68, 20.72]	2025
**Total (95% CI)**			**1342**			**1366**	**100.0%**	**11.93 [6.70, 17.15]**	

Heterogeneity: $Tau^2 = 13.98$; $Chi^2 = 6.59$, df = 2 (P = 0.04); $I^2 = 70\%$
Test for overall effect: Z = 4.48 (P < 0.00001)

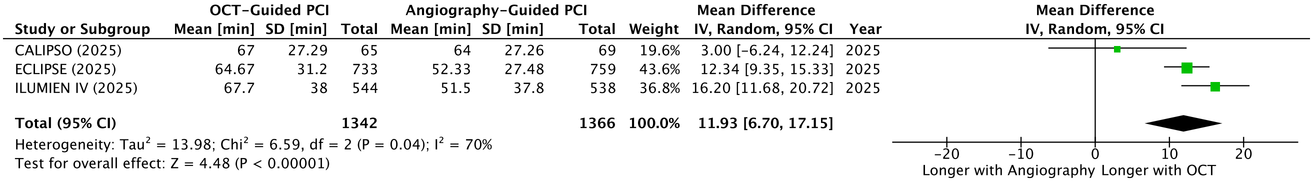

(**f**) Radiation dose

Study or Subgroup	OCT-Guided PCI Mean	OCT-Guided PCI SD	OCT-Guided PCI Total	Angiography-Guided PCI Mean	Angiography-Guided PCI SD	Angiography-Guided PCI Total	Weight	Std. Mean Difference IV, Random, 95% CI	Year
ECLIPSE (2025)	1,354	946.3	733	1,377.67	1,128.2	759	50.8%	−0.02 [−0.12, 0.08]	2025
ILUMIEN IV (2025)	2.02	1.77	544	1.64	1.56	538	49.2%	0.23 [0.11, 0.35]	2025
**Total (95% CI)**			**1277**			**1297**	**100.0%**	**0.10 [−0.14, 0.35]**	

Heterogeneity: $Tau^2 = 0.03$; $Chi^2 = 9.78$, df = 1 (P = 0.002); $I^2 = 90\%$
Test for overall effect: Z = 0.80 (P = 0.42)

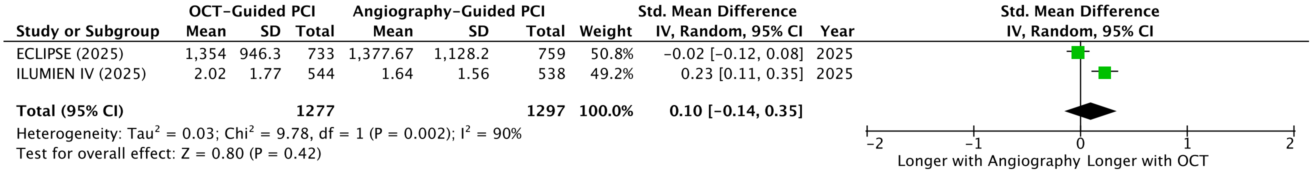

Figure S4. (continued)
